# Investigating Sub-Spine Actin Dynamics in Rat Hippocampal Neurons with Super-Resolution Optical Imaging

**DOI:** 10.1371/journal.pone.0007724

**Published:** 2009-11-09

**Authors:** Vedakumar Tatavarty, Eun-Ji Kim, Vladimir Rodionov, Ji Yu

**Affiliations:** Center for Cell Analysis and Modeling, University of Connecticut Health Center, Farmington, Connecticut, United States of America; CNRS UMR6543 - Université de Nice - Sophia Antipolis, France

## Abstract

Morphological changes in dendritic spines represent an important mechanism for synaptic plasticity which is postulated to underlie the vital cognitive phenomena of learning and memory. These morphological changes are driven by the dynamic actin cytoskeleton that is present in dendritic spines. The study of actin dynamics in these spines traditionally has been hindered by the small size of the spine. In this study, we utilize a photo-activation localization microscopy (PALM)–based single-molecule tracking technique to analyze F-actin movements with ∼30-nm resolution in cultured hippocampal neurons. We were able to observe the kinematic (physical motion of actin filaments, i.e., retrograde flow) and kinetic (F-actin turn-over) dynamics of F-actin at the single-filament level in dendritic spines. We found that F-actin in dendritic spines exhibits highly heterogeneous kinematic dynamics at the individual filament level, with simultaneous actin flows in both retrograde and anterograde directions. At the ensemble level, movements of filaments integrate into a net retrograde flow of ∼138 nm/min. These results suggest a weakly polarized F-actin network that consists of mostly short filaments in dendritic spines.

## Introduction

The majority of the excitatory synapses in central nervous systems are formed onto dendritic spines. Morphologically, dendritic spines appear to be micrometer-sized membrane protrusion from the neuronal dendrites; functionally they serve as compartments for post-synaptic molecules. They come in a variety of shapes [1], most commonly as one of the following: filopodia-like, stubby, mushroom-shaped and cup-shaped. The shape and the size of a spine is determined by the underlying actin cytoskeleton [2], as spines contain a high concentration of filamentous (F-) actin molecules and are mostly devoid of microtubules. In recent years, advanced live cell imaging techniques have revealed that the spines are remarkably dynamic, changing size and shape in a matter of minutes [3]-[5]. These morphological changes are widely believed to affect functional properties of the individual synapses and by extension the neuronal network, and therefore are directly linked to brain's cognitive functions, such as memory and learning. A large body of evidence now exists to support this proposition. For example, many studies have demonstrated changes in spine morphology following electrophysiologically induced long-term potentiation (LTP) or long-term depression (LTD) [6]. Furthermore, a dynamic F-actin cytoskeleton is required for establishing LTP and LTD [7]-[9]. Finally, recent studies in culture showed that the direct application of stimuli to individual spines resulted in an enlargement of the spine and this enlargement required actin [10], [11]. Therefore understanding the actin cytoskeleton is of central importance to the studies of synaptic and neuronal function.

The dynamics of F-actin in spines had been studied with fluorescence redistribution assays. On the whole spine level, fluorescence redistribution after photon-bleaching (FRAP) had been carried out on fluorescently labeled actin to show that it takes about 1 min [12] for the fluorescence signal to recover after the spine is photo-bleached. This recovery was believed to reflect the kinetic dynamics (F-actin turn-over) of the actin cytoskeleton. More recently, two-photon activation was applied to produce fluorescently labeled actin within sub-regions of large spines [13]. Florescence signal activated near the tip of a large spine was shown to redistribute towards the dendritic shaft at the time scale of minutes. This result was attributed to the kinematic dynamics (physical motion of actin filaments, i.e., retrograde flow) of the F-actin. Although these interpretations are intuitive, it should be pointed out that, in principle, fluorescence redistribution experiments do not distinguish kinetic dynamics from kinematic dynamics. For example, fluorescence signal moving away from the tip of the spine could also be attributed to actin monomers depolymerizing from the filament, diffusing to a new location in the same spine, and re-associating with a different filament. Techniques capable of distinguishing these two types of actin dynamics, such as quantitative speckle microscopy (QSM), often provide a greater insight into the organization and mechanism of the actin cytoskeleton [14]. Unfortunately, application of QSM to the studies of dendritic spines faces tremendous technical difficulties, not the least because of the extremely small sizes of the dendritic spines (typically 0.5 – 2 µm [15]), which are quite close to the resolution limit of normal optical microscopes (∼0.3 µm). Comparing to many other dynamic actin structures, such as the leading edge protrusions in migrating cell, much less is understood about the dendritic spines.

The main goal of this paper is to measure both the kinematic and kinetic dynamics of F-actin in dendritic spines at single-filament level with high spatial resolution. To do this, we employed a single-molecule tracking technique based on the photo-activation localization microscopy (PALM) [16], [17]. PALM was originally invented to produce high resolution images of fixed cells. However recent work has extended its applications to tracking single protein molecules in live cells [18], [19]. In PALM imaging, the target protein molecule is labeled with a photo-activatable fluorescent protein, such as EosFP [20]. EosFP is normally a green-fluorescent protein, but can be converted into a red-fluorescent one by blue or ultra-violet (UV) illumination. To carry out PALM image data collection, controlled UV illumination is applied to the sample to stochastically convert one or a few fluorescent protein molecules into the red form. Converted molecules are then imaged with a sensitive microscope as spatially separated single molecules until they photo-bleach. This process can be repeated for many cycles to image many molecules. The advantage of PALM microscopy is that the protein localization accuracy is no longer limited by the diffraction-limited spatial resolution of the microscope. Instead, the well-defined spatial profile of a single-molecule image allows the application of centroid fitting algorithms to determine the positions of the molecules with sub-diffractional resolution [21], [22]. This allows us to monitor the kinematic motions of an F-actin molecule in extremely small distances (> = 30 nm, see results), which is important for cellular structures as small as the dendritic spine. Here we present the quantitative data on the kinematic and kinetic dynamics of F-actin in dendritic spines of hippocampal neurons, and present evidence that actin cytoskeleton in the dendritic consists of mostly short F-actin filaments undergoing very fast turnover.

## Results

### Single-Molecule Assay of F-Actin Dynamics in Lamellipodia

**Figure 1:**
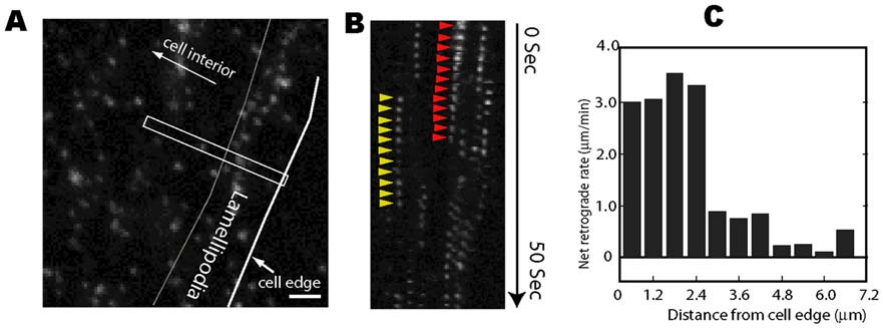
F-Actin cytoskeleton dynamic in the lamellipodia of a Xenopus fibroblast cell visualized with single-molecule tracking. **A.** The fluorescence image of the lamellipodia region showing images of single EosFP-actin molecules. **B.** kymographs (white rectangle area of the left image) of the same cell showing retrograde flow of single EosFP-Actin molecules from the leading edge. Red arrow heads tracked one molecule moving away from the cells leading edge. Molecules deeper inside the cell had a much slower retrograde flow rate. Scale bar represented 2 µm. **C.** The average actin retrograde flow rate versus the distance of the molecule from the edge of the cell (The right-side gray-line in panel A).

To validate our experimental approach, we first applied our technique to examine the actin dynamics in the lamellipodia of Xenopus fibroblast cells (Movie S1, Figure 1) and Xenopus melanophore (Movies S2). Actin dynamics in the lamellipodium region of migrating cells have been extensively studied [23], allowing the comparison of our results with those of earlier studies. We expressed F-actin fused to a photo-activatable fluorescent protein, EosFP, in cultured cells. The fluorescence protein normally emits green fluorescence in the unactivated state, but can be activated by ultra-violet (UV) light to emit red fluorescence. Although there were many EosFP-actin molecules within the cell, they were not detected except for a few molecules that had been activated. New EosFP-actin molecules were repeatedly activated to replenish the molecules photobleached during the previous imaging, allowing the continuous collection of single molecule imaging data. Each image was acquired with 400 ms exposure time. Under this experimental condition, only the signal from F-actin molecules are detected as focused spots; while the signal from monomeric G-actin molecules spreads to a much larger area due to fast diffusion (>8 µm^2^/sec [24]) and is not detected.

Single molecules of F-actin can be clearly seen near the leading edge of the slowly migrating cells (Figure 1A). Within ∼3 µm from the leading edge of the cells, all molecules detected were in sharp focus, indicating that they were within the same focal plane. This was expected because the lamellipodia region is known to be very thin. Further away from the leading edge of the cells, some molecules were slightly blurred (see Movie S1), consistent with the thickness of the lamella regions. The appearance of the PALM-based single-molecule data resembles that of speckle microscopy, which is expected because each individual molecule is a speckle of the smallest possible size. Accordingly, the interpretation of our imaging data is similar to that of speckle microscopy. However, a single molecule can only be from one F-actin filament, thus any movements of the molecule represent the kinematic dynamics of a single filament.

We found that single actin molecules at the lamellipodia continuously move in the retrograde direction at a rate of 2-3 µm/s (Figure 1B), similar to the results from earlier measurements [25], [26]. Furthermore, fast retrograde movements are limited to a region within leading edge of the cell, beyond which the net rate of the retrograde flow is greatly reduced (Figure 1C), indicating a decoupling between the lamellipodium region and the lamella region. In fact, the tracks of individual fast moving molecules in the leading edge region are rarely seen to migrate beyond the first 3 µm from the cell edge into the interior of the cell (Movie S1), indicating that they depolymerize before reaching the lamella region. These observations are consistent with previous findings from quantitative speckle microscopy [14]. Therefore we concluded that our experimental system reported realistic actin cytoskeleton dynamics.

### Kinematic Dynamics of F-Actin in Dendritic Spines

**Figure 2:**
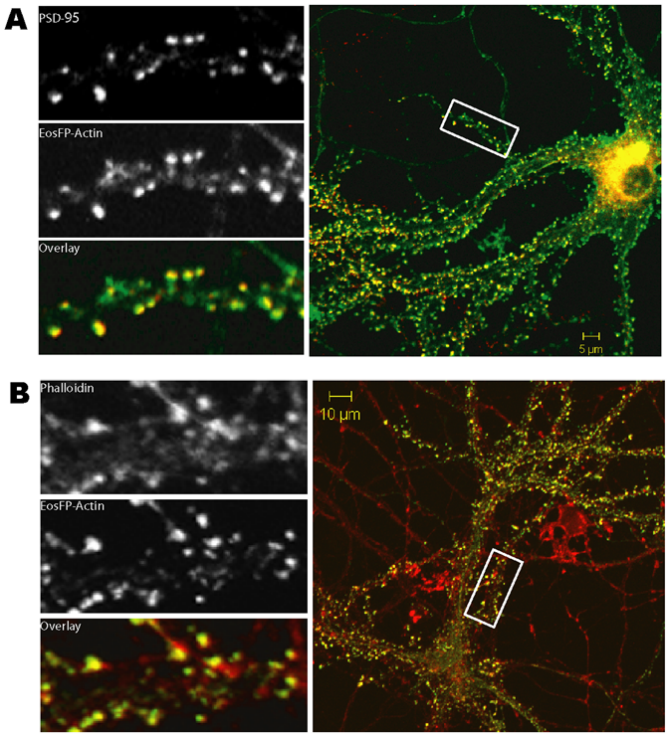
Localization of EosFP-actin in neuron.

We expressed EosFP-actin in hippocampal neurons. As shown in Figure 2, the green fluorescence of EosFP-actin colocalized with endogenous actin molecules, which is highlighted by phalloidin staining (Figure 2A), as well as the post-synaptic marker PSD95 (Figure 2B). These data indicated that the localization of expressed tdEosFP-actin fusion protein corresponds to that of endogenous actin in dendrites and dendritic spines.

**Figure 3:**
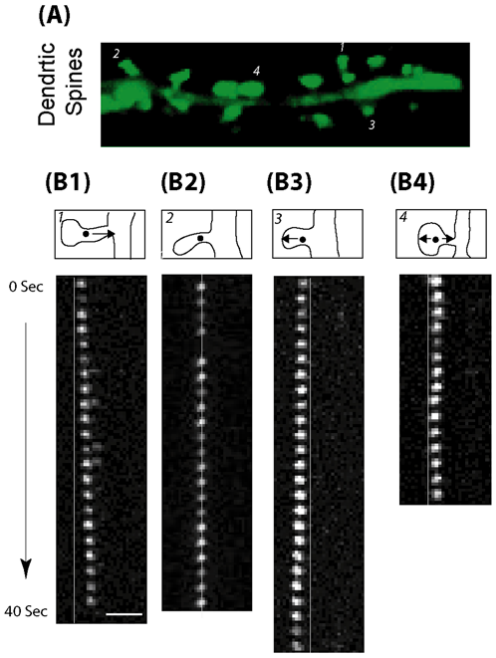
Heterogeneous F-Actin kinematic flow in dendritic spines. **A.** Fluorescence images of dendrites (23DIV) showing mature dendritic spines. **B.** Time-lapse image sequences of single EosFP-actin molecules representing molecules that are moving in retrograde direction (B1), stationary (B2), moving in anterograde direction (B3) and moving randomly (B4). All scale bars are 2 µm.

Unlike in lamellipodia, single-molecule data obtained in dendritic spines exhibited a high level of heterogeneity (Movie S3). We observed at least four different types of F-actin molecules based on their kinematic behaviors. 1. Actin molecules with no detectable motion. Close to half of all molecules belong to this category, making it the dominant category. 2. Actin molecules with vectorial retrograde motion. Although previous results had demonstrated redistribution of actin in the retrograde direction (i.e., towards the dendritic shaft), it has not been directly shown before that that a vectorial flow of the filaments contributes to such redistribution. 3. Actin molecules with vectorial anterograde motion. These molecules move into the spine away from the dendritic shaft. Although the number of molecules in this category is small, this type of kinematic dynamics is completely unexpected. Both the retrograde moving molecules and the anterograde moving molecules seem to coexist in the same spine (see for example, Movie S4), which indicates that the direction of actin flow is not dependent either on the specific spine or on the existing physiological state of the spine 4. Finally, molecules with random-walk type of motion. Examples of each type of molecule are shown in Figure 3A and 3B.

Most of the filament movements we observed in spines are over short distance, in the range ∼30 - 200 nm. To seek evidence that these small movements reflect intrinsic cytoskeleton dynamics, we tested the effect of actin-perturbing drug jasplakinolide on the neuron cells. Jasplakinolide inhibits actin depolymerization [27], which results in a loss of G-actin, hence inhibiting F-actin turnover. We found that the drug reduces and eventually stopped kinematic movements of all types completely within minutes (Movie S5), confirming that the observed kinematic movements requires the integrity of actin cytoskeleton.

**Figure 4:**
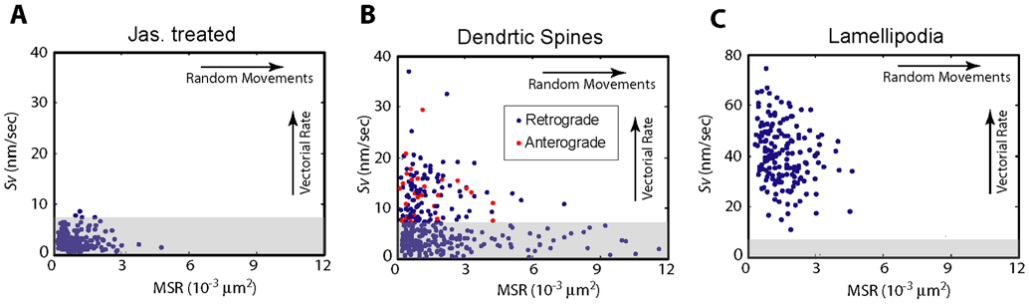
Quantification of the F-actin kinematics in dendritic spines. (**A**) 2D scatter plot of the Sv and MSR values from molecules in neuron after treatment with jasplakinolide. (**B**) 2D scatter plot of the Sv and MSR values from molecules in dendritic spines. Each dot represents one actin molecule. The open circles represent molecules with a net motion in anterograde direction. (**C**) 2D scatter plot of the Sv and MSR values from molecules in the lamellipodia region of the Xenopus fibroblast. Note the different scale in the y-axis. Shaded area denotes molecules that showed little or no vectorial movements (Sv <7.5 nm/sec).

Figure 4 summarizes the quantification of F-actin kinematics dynamics in several different systems. Due to the complex kinematic dynamics of F-actin, simple parameters, such as average retrograde flow rate, have ambiguous physical meanings. Therefore, we applied a linear regression model to analyze individual single-molecule trajectories that are >12 s in duration: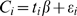, where C is the coordinates of the molecule, t is the time and *ε* is the residue from the least square method. The results provided two parameters for each molecule: the slope of the regression model S*_v_*, and the mean square residues (*MSR*) – from each molecule analyzed, defined as:
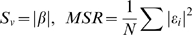



Therefore, the S*_v_* value measures the rate of the vectorial movement, and the *MSR* value measures the amplitude of the random motion of the molecule. The real experimental measurements of molecular positions have limited accuracy (see methods); the measurement errors also contributed to the value of *MSR*, as well as introducing noise to *S_v_*. Computer simulation further confirms the interpretation of these parameters (Figure S1). Scatter plots of *S_v_* and *MSR* values (Figure 4A-C), in which each point plotted represents one F-actin molecule analyzed, allowed for a visual comparison of actin kinematics in different conditions.

Figure 4A shows the results from jasplakinolide treated neuron cells. Under this condition, the molecules are expected to be stationary, and therefore have both small *S_v_* values and small *MSR* values. The result shown in Figure 4A confirms that prediction and agrees well with computer simulation of stationary molecules (Figure S1). Figure 4B shows the analysis of ∼270 actin single-molecules tracks obtained in dendritic spines. Their *S_v_* values and *MSR* values spread out over a large range, consistent with the high degree of heterogeneity observed. For molecules with *S_v_*>7.5 nm/sec – an empirical threshold based on Figure 4A – we further analyzed the directions of their vectorial movements. We found 27 molecules (10% of all measurements), denoted as open circles, in anterograde direction, while the rest (34% of all measurements), denoted in closed circles, in retrograde direction. Among molecules with *S_v_* <7.5 nm/sec, some have large *MSR* values, indicating random movements of the molecules. About 37% of molecules were considered stationary (*S_v_* <7.5 nm/sec; *MSR *<0.002 µm^2^), as their parameters were indistinguishable from molecules in Figure 4A. As a comparison, the data from melanophore lamellipodia are shown in Figure 4C, in which all molecules were moving vectorially in the retrograde direction. The narrower distribution of *MSR* in these cells indicates a more or less uniform retrograde flow. Therefore, our results and analysis demonstrated a complex actin dynamics in dendritic spines that is much more heterogeneous than seen in the lamellipodia.

### Net Retrograde Flow of F-Actin in Dendritic Spines

**Figure 5:**
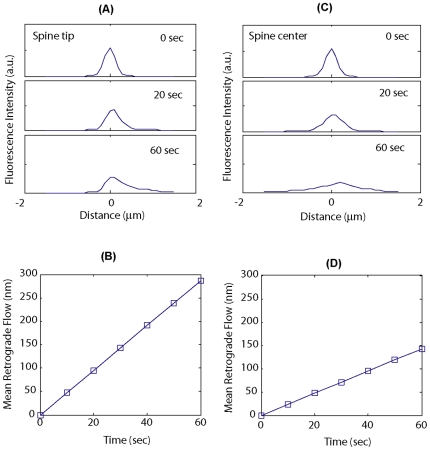
F-actin redistribution in the dendritic spine modeled on single-molecule kinematics measurements. (**A**) The redistribution of a pool F-actin initially at a spine tip over one minute time span. The initial spread of the molecules is assumed to be diffraction-limited (full-width at half-maximum (FWHM)  = 300 nm). The intensity profile evolves over time, calculated according to the distribution of *Sv* values from single-molecule measurements. (**B**) Calculated net retrograde flow of F-actin based on the simulated data in A. (**C**) The redistribution of a pool F-actin initially at the center of a spine over one minute time span. (**D**) Calculated net retrograde flow of F-actin based on the simulated data in C. See text for the details.

The filaments moving in retrograde direction outnumbers those in anterograde direction, therefore, on average, F-actin filaments in dendritic spines flows in a net retrograde direction. This conclusion is in agreement with a previous measurement based on a two-photon fluorescence redistribution assay. In order to carry out a quantitative comparison between our results with the previous data, we performed computer simulation to mimic the fluorescence redistribution experiments (Figure 5). In the fluorescence redistribution experiment, PA-GFP labeled actin is expressed to fill the neuron cell. A focused laser beam illuminating at the tip of a spine activates a small pool of labeled actin, whose fluorescence signal is then followed with time-lapse imaging. The spatial redistribution of the fluorescence signal, therefore, reflects spatial redistribution of the actin molecules over time. In our simulation, a pool of actin filaments was initially distributed within a small region, according to a Gaussian-shaped probability distribution function with a full-width-at-half-maximum (FWHM) of 300 nm. This condition mimics the initial pool of photo-activated molecule by a focused laser. The individual filaments were then allowed to move with a distribution of rates according to the measured distribution of *Sv* values from our experiments. Because the position of each actin molecule is tracked in the simulation, the spatial profile of actin concentration can be followed over time (Figure 5A). As different molecules move at different speed, the calculation predicts a significant broadening of the actin profile, resulting in an asymmetric shaped distribution with a longer tail towards the retrograde direction. Consequently, the center-of-the-mass of the actin pool moves towards the retrograde direction too. These qualitative features agrees perfectly with the previous experimental measurements [13], confirming the validity of our single-filament kinematics data. Quantitatively, the position of the center-of-the-mass of the actin pool can be calculated over time (Figure 5B), and fit with a linear function to obtain the net retrograde flow rate. For our simulation, the net retrograde flow rate is 138 nm/min, which is within the range previous reported (∼50 – 1200 nm/min [13]) by the fluorescence redistribution assay, but at the lower end. One possible reason for this is the difference in the size of the spines investigated. Due to the constraints of the diffraction-limited spatial resolution, the fluorescence redistribution experiments were most reliable when the spine is very large. In fact, the large flow rates were only seen in the largest spines [13]. Furthermore, in the experiment, part of the fluorescence redistribution could arise from molecules dissociating from their original filaments and free-associating with new filaments in the same spine, especially for bigger spines. Since this type of redistribution is not due to true kinematic flow, it is not taken into account in our simulation. Therefore, the fluorescence redistribution experiments probably over-estimated the true retrograde flow rate.

We also calculated the redistribution of a pool of F-actin initially situated at the center of the spine (Figure 5C). In this case, the broadening of the spread is more significant and the net retrograde flow diminished to 75 nm/min (Figure 5D), due to a larger contribution from the anterograde fraction of the molecule. Unfortunately, experiment has not been performed under this condition.

### Kinetic Dynamics of F-Actin in Dendritic Spines

We sought to understand why a large portion of filaments were stationary. In some actin systems, the kinematic dynamics is correlated with kinetic dynamics, i.e., fast turnover of the F-actin filament lead to kinematic movements of the filament, and kinematically stationary filaments represents ones that are capped. Another possibility is that the filaments' lengths are much smaller than the dimension of the whole spine, in which case kinetic turnover could result in treadmill without resistance, and would not lead to kinematic motion of the actin units in the filaments. Therefore we set out to measure the turnover kinetics of the F-actin in our experimental system.

The turnover kinetics of F-actin can be evaluated with two methods. First, we examine the lengths of single molecule tracks. The kinetic dynamics of the filament is manifested in the time duration for which a molecule could be tracked. Positions of a photo-activated EosFP-actin molecule can be followed until the molecule is depolymerizes and diffuses away. Therefore, a faster turnover rate is correlated with shorter durations of the tracks; thus the average length of the tracks is a relative measure of how fast the turnover kinetics are. Quantitative relationship between the turnover rate and the tracking length, however, is complicated by two factors. First, photobleaching generally shortens the tracking length and causes overestimation of the kinetic turnover rate unless the true filament turnover rate is much faster than the photobleaching rate. Second, the tracking often starts with the photo-activation of a random F-actin molecule, which could be sitting at any place on a filament, and a distribution of tracking lengths could be obtained from the same turnover rate. Nevertheless, the average single-molecule track length should provide a relative measure of the turnover kinetics. For example, if filament turnover is fast, the characteristic length of the single-molecule trajectories would be significantly shorter than what would be expected based on their photo-bleaching rate. Alternatively, if the filament is capped or turnover is very slow, the lengths of the single-molecule trajectories would be longer, equivalent to their rates of photo-bleaching.

**Figure 6:**
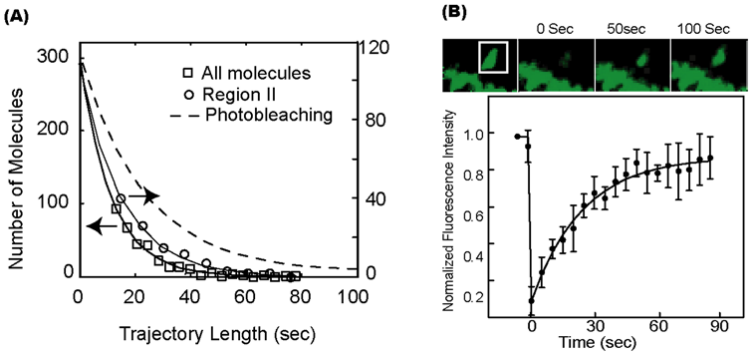
Kinetic dynamics of F-actin in dendritic spines. (**A**) Distributions of the lengths of single-molecule tracks from dendritic spines. The solid line is the single exponential fit. The dashed line is the expected distribution from only photo-bleaching. The distribution for the subpopulation excluding stationary filaments is also plotted as comparison. (**B**) FRAP measurements in dendritic spines. Top images show bleaching and recovery of a spine. The bottom graph shows the average normalized fluorescence intensity of 10 spines measured. Errorbars represent standard deviation. The solid line is the fit with single exponential recovery.

Plotted in Figure 6A is the distribution of the track lengths from dendritic spines. This distributions can be fitted with a single exponential function to obtain a time-constant of *τ* = 12.5 s, which is significantly shorter than the photo-bleaching time of *τ*_pb_ = 24.2 *s* (see methods and Figure S2). Therefore, in average it takes ∼12.5 sec for any randomly photoactivated molecule to reach the pointed ends of the filament and to be depolymerized.

The turnover rates can also be examined with fluorescence redistribution after photo-bleaching (FRAP) experiments (Figure 6B). The fluorescence recovery curve could be fit well with an exponential function (Figure 6B). The time constant for the recovery was |  = 30.6±5 s (n = 10), corresponding to the time required to exchange F-actin in the whole spine. The immobile fraction corresponds to a small part (9.1%) of the total actin intensity, indicating that there were few capped filaments. These numbers agree well with earlier FRAP measurements [12] using GFP-actin as the fluorescent probe. The difference between the FRAP experiment and the single-molecule tracking experiment is that the FRAP experiment measures the turnover time of whole spine, while the single-molecule experiment measures the turnover time of individual filaments. It is also interesting that the turnover time of whole spine is significant longer than the turnover time of individual filaments, which suggests that an actin molecule dissociated from one filament would have a high probability to re-associate with another filament before escaping the spine.

The above measurements indicated that >90% of filaments were undergoing fast turnover; however not all the kinetic turnovers were translated into kinematic movements of the filaments, suggesting that F-actin in dendritic spines were made of mostly short filaments.

## Discussion

In this paper we demonstrated a single-molecule imaging assay for analyzing the kinematic dynamics of F-actin in dendritic spines. The method is general enough so that it should be useful for other small cellular compartments. Studying actin kinematics provides clues to the underlying cytoskeletal organization. We argue that at least three mechanisms can give rise to kinematic movements. First, elongation of an F-actin filament from the barbed end, when reaching resistive barrier, will generate force to push filament back. Movement of this type is along the orientation of the filament and towards the pointed end. Secondly, nucleation and/or growth of other filaments could run into and push the filament under observation, causing it to move. In this case, the movement does not need to follow the direction of the filament. Finally, force generating molecular motors, such as myosin, could also produce movements of F-actin. In our case, jasplakinolide stabilized F-actin shows no sign of kinematic movements, which seems to suggest the first two mechanisms. Although a better test of the effects of motor proteins is to chemically inhibit motor activities, unfortunately, the drug commonly used for this purpose, blebbistatin, is a fluorescent molecule itself and thus strongly interfere with single-molecule detection. Therefore we were unable to perform this test. On the other hand, the existence of many randomly moving molecules cannot be accounted for by the first mechanism. It is likely that the second mechanism plays an important role in spines.

**Figure 7:**
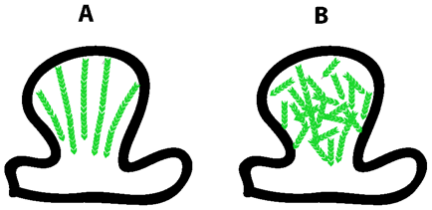
Models of actin organization in dendritic spines. (A) Highly polariozed actin cytoskeleton. Actin forms long filaments with the barbed ends pointing towards the tip of the spine, where the polymerization rate is high. This model is inconsistent with the experimental results. (B) Weakly polarized actin cytoskeleton. Most actin filaments are short and not well aligned. Barbed ends are distributed all over the spine. Polymerization and filament growth could happen everywhere. This model is more consistent with the experimental results. See text for the details.

While at the ensemble level, a slow retrograde flow could be observed in dendritic spines, we were surprised to find that that at the individual filament level, the kinematic dynamics of F-actin in dendritic spines are highly heterogeneous. In particular, it is intriguing to see that the fast kinetic turnover of F-actin has only driven a relatively small portion of filaments to flow in retrograde direction. This observation suggests that actin filaments are short in dendritic spines. Consider two models of actin organization shown in Figure 7. In one model (Figure 7A), a highly polarized actin cytoskeleton is made of long filaments of actin. In this case, actin polymerization and filaments growth happen predominantly at the tip of the spine, therefore one would expect most of the filaments to move in retrograde direction. Such a model is analogous to F-actin in lamellipodia, but is inconsistent with our experimental findings. On the other hand, if actin cytoskeleton is made of very short filaments (Figure 7B), the growth of many filaments will not face resistance and therefore will not generate retrograde force. Furthermore, as the barbed ends are distributed through out the spines, filaments growth is not limited to the tip region of the spine. The polymerization and growth of the filaments could push other surrounding filaments, leading to a complex force generation mechanism. The growth of filaments near the neck of the spine, for example, could push other filaments up towards the tip, resulting in the anterograde flows that we observed in some cases. Therefore, the model shown in Figure 7B is more consistent with our experimental results. Overall, the level of complexity in dendritic actin cytoskeleton demands a comprehensive approach that combines single-filament measurements with stochastic modeling, which should be a focus of our future research.

## Methods

### Cell Culture, Plasmids and Transfection

The vector carrying dimer EosFP coding sequence was a generous gift from Dr. Wiedenmann (University of Ulm). The human actB cDNA sequence was amplified with PCR from a cDNA plasmid (OpenBiosystems, accession number BC004251), which was part of the IMAGE cDNA clone collection and inserted at C-term of the EosFP gene.

Embryonic hippocampal neuron cultures were prepared from E17 Sprague–Dawley rat (Charles River Laboratories). Cells were plated on glass bottom culture dishes that were thoroughly cleaned by sonicating sequentially in 10% HCl, 20% NaOH and Millipore water and coated with poly-L-lysine (Sigma) overnight before use. In some cases cells were transfected by nucleofection (Amaxa-Germany) with EosFP-actin plasmid DNA (3-4 µg) before seeding at low-to-medium density. Cells were allowed to recover for 3 hrs in DMEM. After 3 hrs DMEM was replaced by Neurobasal media containing supplements. Cell cultures were maintained for up to 25 DIV in Neurobasal medium (Gibco) supplemented with B27 before switching to Hibernate E media (BrainBits) before the microscopy experiment. For FRAP experiments on filipodia and spines Lipofectamine 2000 (Invitrogen) was used to transfect neurons 8-16 DIV according to manufacturers recommendations.

Xenopus embryonic fibroblast cells, and Xenopus melanophore was transfected via microinjecting plasmid DNA into the cells 24 hours prior to imaging. Before imaging the cells were transfered to hibernate E media.

To inhibit actin filament dynamics cells were incubated in Hibernate E supplemented with 1 µM Jasplakinolide (Invitrogen) (stock at 1 mM dissolved in methanol and kept at -20°C).

### Immunostaining

Hippocampal neurons 20-22 DIV nucleofected with EosFP-Actin during plating were fixed in 4% paraformaldehyde in PBS for 20 minutes. Cells were washed 3 times (5 mins.)in PBS and incubated for 20-25 min in a solution containing 10% horse serum, 0.125% TritonX-100 in PBS. Next the cells were incubated with either mouse αPSD-95 antibody (1∶500) (a generous gift from Dr. Matt Rasband, Baylor college of Medicine, TX) or stained with Texas red labeled phalliodin (Invitrogen) for 1 hr and washed 3 times in PBS. Cells stained with mouse αPSD-95 antibody were incubated with α-mouse Alexafluor 647 secondary antibody (1∶400) (Molecular Probes) for 1 hr. Cells were fixed in Prolong Gold antifade (Invitrogen) and imaged under a Zeiss 510 confocal microscope with a 63×1.4 N.A. Oil immersion objective.

### Single-Molecule Microscopy

Single-molecule fluorescence images were taken with a modified epi-fluorescence microscope (Olympus IX81) equipped with 60x microscope objective (NA = 1.45, Olympus) and a TE-cooled EM-CCD camera (PhotonMax, Roper Scientific, Trenton, NJ). To image single-molecules with PALM microscopy, a 405 nm diode laser (Cube laser system, Coherent Inc., Santa Clara, CA) was the light source for photo-activation. The activation pulse was controlled controlled by the computer through a D/A conversion interface (NI-USB-6251, National Instruments, Austin, TX). The green fluorescence of unactivated EosFP was excited with the 488 nm laser line from an argon ion laser (Melles Griot). For single-molecule imaging a 532 nm DPSS diode laser (Lambda) was used for excitation at a power density of ∼0.4 kW/cm. Unless otherwise stated, single-molecule time-lapse images were taken at 2 sec interval with 400 ms acquisition time for each image. Image acquisition software was built on top of the μManager platform (http://micro-manager.org).

### Image Analysis

Single molecule trajectories (positions versus time) were constructed using standard particle tracking algorithm. From each data set, a region of interest that included only the dendritic spine region was manually defined based on the green-fluorescence signal. Single-molecule trajectories outside this region of interest was excluded from the dataset. So was trajectories shorter than 12 sec. Further analysis of the single molecule trajectories was carried out in Matlab.

### FRAP and Confocal Microscopy

Neurons were transfected with the actin-Eos construct using lipofectamine2000 at 14-21DIV to observe actin dynamics in spines. 24-48 hours after transfection neurons were medium changed to low fluorescence hibernate E buffer and observed under a Zeiss LSM510 meta confocal microscopy. Spines were observed with a 40X, 1.2 numerical aperture(NA) water-immersion objective. All data was collected at room temperature. 20 frames were scanned at 5sec interval with fully opened pinhole. To photo-bleach spines 488nm laser was concentrated on a area of 1 µm×2 µm area (10 iterations). Images were collected before and after bleaching using low laser intensities to reduce bleach while monitoring and recovery was monitored for 2 min.

### EosFP-Actin Photobleaching Kinetics

Single-molecule photobleaching kinetics of EosFP-actin is measured in xenopus fibroblast cells treated with jasplakinolide (see Figure S2). The actin molecules on stress fibers after the cells were treated are extremely stable and provide a good way to measure the photobleaching kinetics under imaging conditions that is close to those in the the neuronal cell experiments. We analyzed more than 800 single molecule trajectories to determine the time it took to photobleach single molecules. The histogram is best-fitted with a double exponential decay function, with the first decay time constant τ_1_ = 12.3 imaging frames (24.6 sec) accounting for 97% of the molecules. A much smaller fraction (3%) of the molecules had a much slower photobleaching kinetics with a time constant τ_2_ = 66.1 frames. Such a long tail in the photobleaching kinetics is common for fluorescent protein molecules. This second slow-bleaching population is particularly obvious in jasplakinolide treated cells and easy to see in time-lapse movies because they lasted for such a long time, but are almost never seen in the untreated neuronal cells, because the turnover of the filament is almost always faster.

### Resolution of Measuring Single-Molecule Centroid Positions

We determined the resolution of single-molecule centroid positions by imaging EosFP-actin in neurons treated with jasplakinolide. Positions of individual actin molecules were repeated measured by the PALM microscope in 2 s intervals. The fluctuation in the measured positions were recorded and the distribution was plotted (Figure S2). The distribution fit well with the theoretical prediction of probability density:
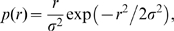
assuming a symmetrical 2D random noise that is normally distributed. The parameter from the fitting indicated that the error in positional measurements was 28.5 nm. The average contribution of the measurement error to *MSR* is:
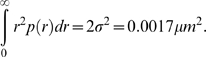



## Supporting Information

Figure S1 2D scatter plot of the Sv and MSR values from computer-simulated single-molecules trajectories. A1. Simulation of stationary molecules. The spread is due to measurement error. A2. Molecules under random walk with an equivalent diffusion constant of 100 nm2/s. The random motions mostly push the distribution into region III, but also “leaks” into region II because of the limited trajectory lengths. A3. Molecules undergoing only vectorial movement at 20 nm/s and without random motions. The distribution is clustered in region II.
(0.24 MB TIF)

Figure S2 Characterization of the EosFP-actin single-molecule property using jasplakinolide stabilized stress fibers. A. Fluorescence image of a Xenopus fibroblast cell after jasplakinolide treatment showing clear stress fiber structures. B. A single molecule image of the boxed region in A. C. Histogram of the length of single-molecule trajectories before photo-bleaching. The data was fitted with double exponential function. D. Histogram of single-molecule positional measurement error obtained by repeatedly measuring the position of the same actin molecule on the stress fiber.
(0.36 MB TIF)

Movie S1 Retrograde flow of actin network in lamellipodia region of a Xenopus fibroblast cell, visualized by single actin molecule imaging. The movie was sped up 40 times from real time.
(1.85 MB AVI)

Movie S2 Retrograde flow of actin network in lamellipodia region of a Xenopus melanocyte cell, visualized by single actin molecule imaging. Beginning of the movie shows shows the overall fluorescence signals from the activated actin molecules. The red traces were the trajectories of individual molecules. The movie was sped up 80 times from real time.
(10.32 MB AVI)

Movie S3 Single actin molecules in dendritic spines showing vectorial movements of various directions. The left side image shows the green fluorescence signal. The yellow pointers indicate retrograde moving molecules; the green pointers indicate anterograde moving molecules; and the red pointers indicate randomly moving molecules. The movie was sped up 40 times.
(4.97 MB MOV)

Movie S4 Actin molecules in dendrites and spines after jasplakinolide treatment. Almost all movements stopped. The movie was sped up 80 times.
(1.95 MB AVI)

Movie S5 Bidirectional movements of two actin molecules in the same spine. The left side fluorescence image shows the morphology of the dendritic process. The movie was sped up 20 times.
(0.76 MB AVI)
