# Supplementary figures and images for: Investigating Sub-Spine Actin Dynamics in Rat Hippocampal Neurons with Super-Resolution Optical Imaging
Source: PLoS One. 2009 Nov 9;4(11):e7724. doi: 10.1371/journal.pone.0007724 (PMC2771285; doi:10.1371/journal.pone.0007724)

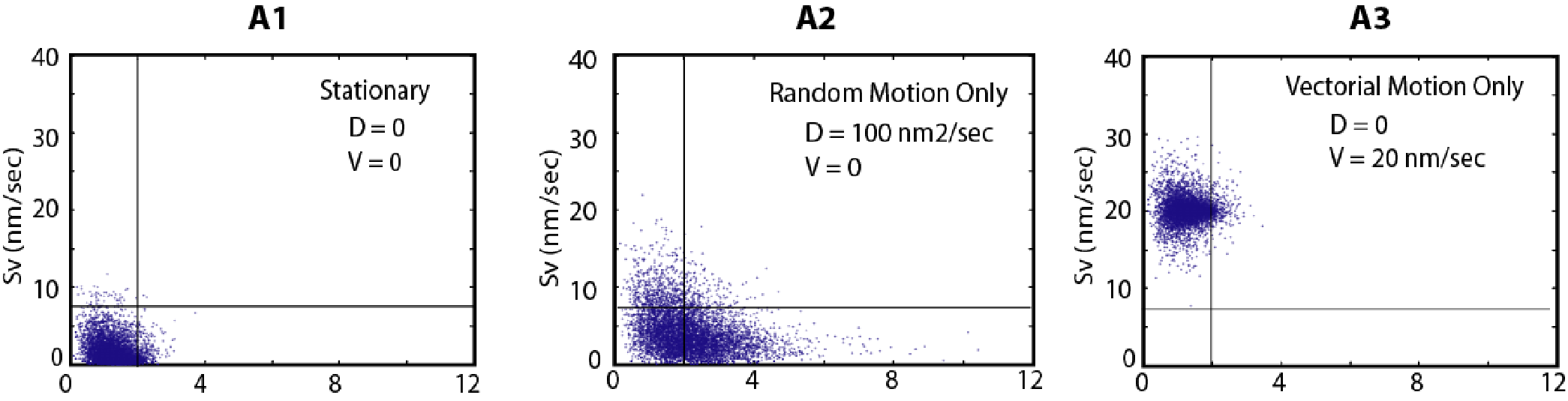

Supplement: Figure S1 — 2D scatter plot of the Sv and MSR values from computer-simulated single-molecules trajectories. A1. Simulation of stationary molecules. The spread is due to measurement error. A2. Molecules under random walk with an equivalent diffusion constant of 100 nm2/s. The random motions mostly push the distribution into region III, but also “leaks” into region II because of the limited trajectory lengths. A3. Molecules undergoing only vectorial movement at 20 nm/s and without random motions. The distribution is clustered in region II. (0.24 MB TIF) [file pone.0007724.s001.tif]

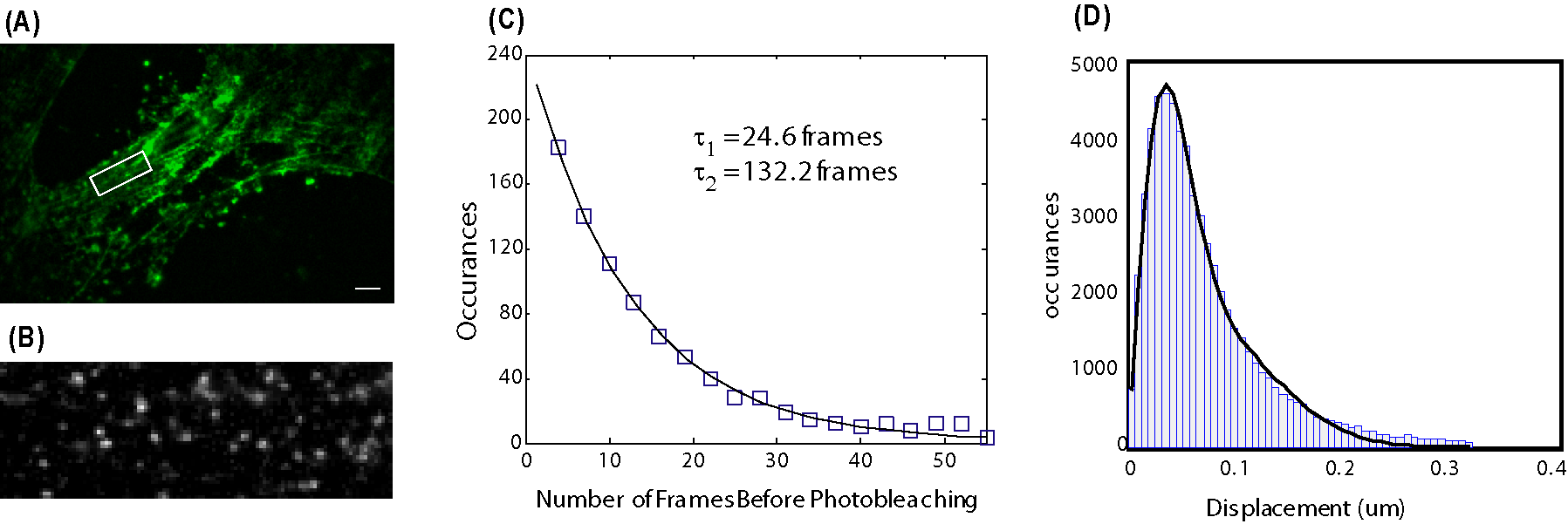

Supplement: Figure S2 — Characterization of the EosFP-actin single-molecule property using jasplakinolide stabilized stress fibers. A. Fluorescence image of a Xenopus fibroblast cell after jasplakinolide treatment showing clear stress fiber structures. B. A single molecule image of the boxed region in A. C. Histogram of the length of single-molecule trajectories before photo-bleaching. The data was fitted with double exponential function. D. Histogram of single-molecule positional measurement error obtained by repeatedly measuring the position of the same actin molecule on the stress fiber. (0.36 MB TIF) [file pone.0007724.s002.tif]
